# Clearance of penicillin allergies via direct oral provocation testing (DOPT): a systematic review

**DOI:** 10.1017/ash.2025.10080

**Published:** 2025-07-31

**Authors:** Michael Dore, Ashley Otto, Annie Wang, Carly Heintz, Sarah Cantrell, Daniel Shields, Allyson Burkhart

**Affiliations:** 1 Duke University School of Medicine, Durham, NC, USA; 2 Durham Veteran’s Affairs Medical Center, Durham, NC, USA; 3 Uniformed Services University of the Health Sciences, Bethesda, MD, USA; 4 Naval Medical Center Portsmouth, Portsmouth, VA, USA

## Abstract

**Objective:**

Penicillin allergies are reported in 10–15% of the US population, but the actual rate is less than 1%. Inappropriate penicillin allergies are associated with adverse patient outcomes, poor antimicrobial stewardship, and increased healthcare costs. Direct oral provocation testing (DOPT) is a safe and cost-effective way to remove false penicillin allergy labels (PAL). However, widespread implementation is currently limited due to inadequate safety data and protocol variations. This systematic review evaluates the safety of single-dose, nongraded DOPT by the nonallergist.

**Design:**

Systematic review. MEDLINE, Embase, Web of Science, and the Cochrane Central Register of Controlled Trials were searched from inception to May 2025.

**Setting:**

Inpatient (Intensive care unit (ICU) and general medical ward) and outpatient

**Participants:**

Adults with self-reported penicillin allergies deemed low risk by a validated scoring system.

**Interventions:**

DOPT by nonallergists with single-dose oral amoxicillin 250 mg with a 60-minute observation period.

**Results:**

3 352 studies were identified, 15 were included in the analysis. Of the 1786 patients who completed DOPT, 66 (3.7%) experienced any reaction: 27 (1.5%) immediate rashes, 24 (1.3%) delayed rashes, and 15 (.8%) other reactions. No cases of anaphylaxis, angioedema, or epinephrine use were reported.

**Conclusion:**

The use of single-dose DOPT in patients deemed low risk, using a validated risk scoring tool, is safe, with low rates of mild reactions and no serious adverse events. A nonallergist can significantly improve penicillin delabeling rates and patient outcomes using this approach.

## Introduction

**Figure 1.**
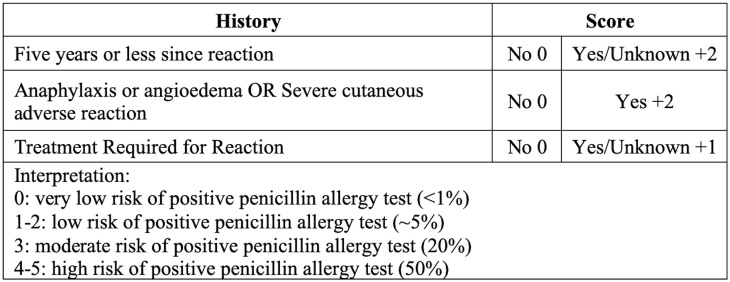
PEN-FAST^13^.

**Figure 2.**
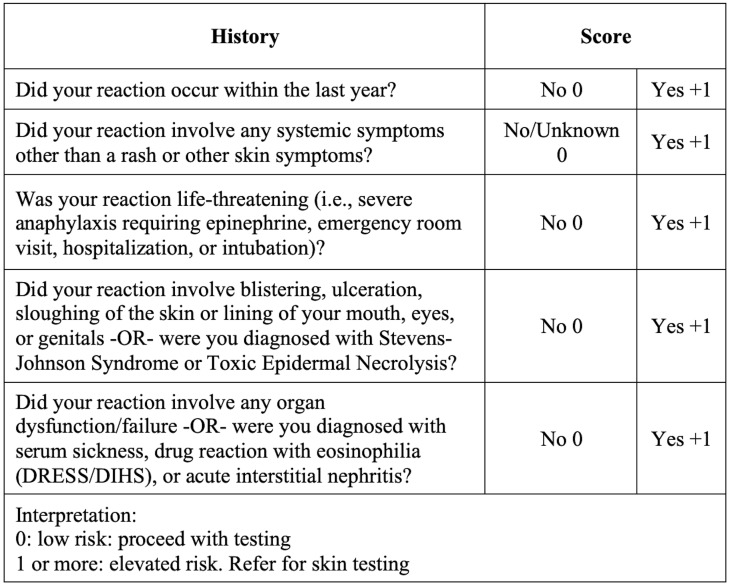
PARSS^5^.

Approximately 15% of hospitalized patients and 10% of the general United States population report a penicillin allergy. However, the actual immunoglobulin-E (IgE)-mediated allergy rate is less than 1%.^1^ This discrepancy in allergy label rates is associated with significant health consequences, including inappropriate use of broad-spectrum antibiotics, prolonged hospital stays, elevated healthcare costs, a 50% increased risk of postoperative infection, and a 14% increased risk of all-cause mortality.^2,3^ Historically, penicillin allergy delabeling has been performed by allergists through a two-step process: a skin test followed by oral provocation testing.^1,4^ Recent evidence has demonstrated the safety and cost-effectiveness of single-dose direct oral provocation testing (DOPT) in select patients when combined with a scoring tool based on clinical history of the allergic reaction such as the Penicillin Allergy Decision Rule (PEN-FAST; Figure 1) and the Penicillin Allergy Risk Stratification Score (PARSS; Figure 2).^1,4,5^ A patient can be considered low risk if they have a score of 2 or less using PEN-FAST or a score of 0 using PARSS.^4,5^

Given the high prevalence of reported penicillin allergies, low rate of true penicillin allergies, and the benefits to the individual and health care system, nonallergist-initiated delabeling is recommended by multiple medical professional organizations but has never been widely implemented. One barrier to widespread implementation is lingering safety concerns, potentially due to published studies with small sample sizes. Another potential barrier is protocol variability. Numerous DOPT protocols have been published with varying doses (ie, amoxicillin 250 mg vs 500 mg) or the number of doses (ie, a grade challenge). There are currently no randomized controlled trials comparing the different DOPT. Systematic reviews by Blumenthal, Cooper, and Powell highlight these heterogeneities in protocols while finding insufficient evidence to recommend one protocol over another.^6–8^ To date, no systematic reviews have examined a single-dose DOPT. We present a systematic review to address these limitations by evaluating the safety of single-dose (amoxicillin 250 mg), nongraded DOPT performed by nonallergists in low-risk patients.

## Methods

This work is a systematic review. It was carried out using the Cochrane Handbook for Systematic Reviews of Interventions and was reported following the PRISMA 2020 statement: an updated guideline for reporting systematic reviews.^9^ The protocol was registered on PROSPERO (2023 CRD42023423873). Available rom: https://www.crd.york.ac.uk/prospero/display_record.php?ID=CRD42023423873

### Eligibility criteria

Inclusion criteria were: (1) adults aged 18 or older with reported penicillin allergy in either inpatient or outpatient settings; (2) allergy history deemed as low risk based on a validated scoring system such as PEN-FAST or PARSS; and (3) DOPT performed with a single-dose of oral amoxicillin (250 mg) followed by a 60-minute observation period. Exclusion criteria were: (1) pregnancy; (2) pediatric-aged patients (<18 yr of age); (3) skin testing before DOPT; (4) graded oral challenge; or (5) amoxicillin dose other than 250 mg.

### Data sources and search strategy

MEDLINE (via Ovid), Embase (via Elsevier), Cochrane Library Central Register of Controlled Trials (via Wiley), and Web of Science—SCI Expanded and SSCI (via Clarivate) were searched from database inception to May 2025.

A professional medical librarian (SC) developed and conducted the search strategy with input on keywords from the other authors. The search strategies included database-specific controlled vocabulary terms and keywords searched in the titles and abstracts for the following concepts: penicillin and derivatives, allergy, and direct oral challenge. The searches were independently peer-reviewed by a librarian using a modified PRESS Checklist and validated against preselected articles. The original searches were conducted on May 8, 2023. A search update across all databases was conducted on May 5, 2025, to identify newly published studies. The full, reproducible search strategies for all included databases are in the Appendices and available online (http://hdl.handle.net/2193/TH83M010F ).

All citations were imported into Covidence, a systematic review screening software (Covidence systematic review software, Veritas Health Innovation, Melbourne, Australia. Available at www.covidence.org.

### Selection process

Two reviewers screened articles at the title and abstract level, two reviewers at the full-text level, and two reviewers at the data extraction level using Covidence systematic review screening software. At each level, any conflicts were resolved by consensus. Study types included randomized controlled trials and prospective cohort trials, while published articles or abstracts were limited to those in the English language. Studies were excluded if they did not meet the eligibility criteria. The investigator was contacted if data were unavailable in the published study.

### Data items

The primary outcomes were: 1) severe adverse reactions defined as anaphylaxis, angioedema, any respiratory distress requiring intubation, or treatment with epinephrine, and 2) any reaction following DOPT.

### Study risk of bias assessment

The quality of the included studies was assessed using the JBI Critical appraisal tool for cohort studies.

## Results

**Figure 3.**
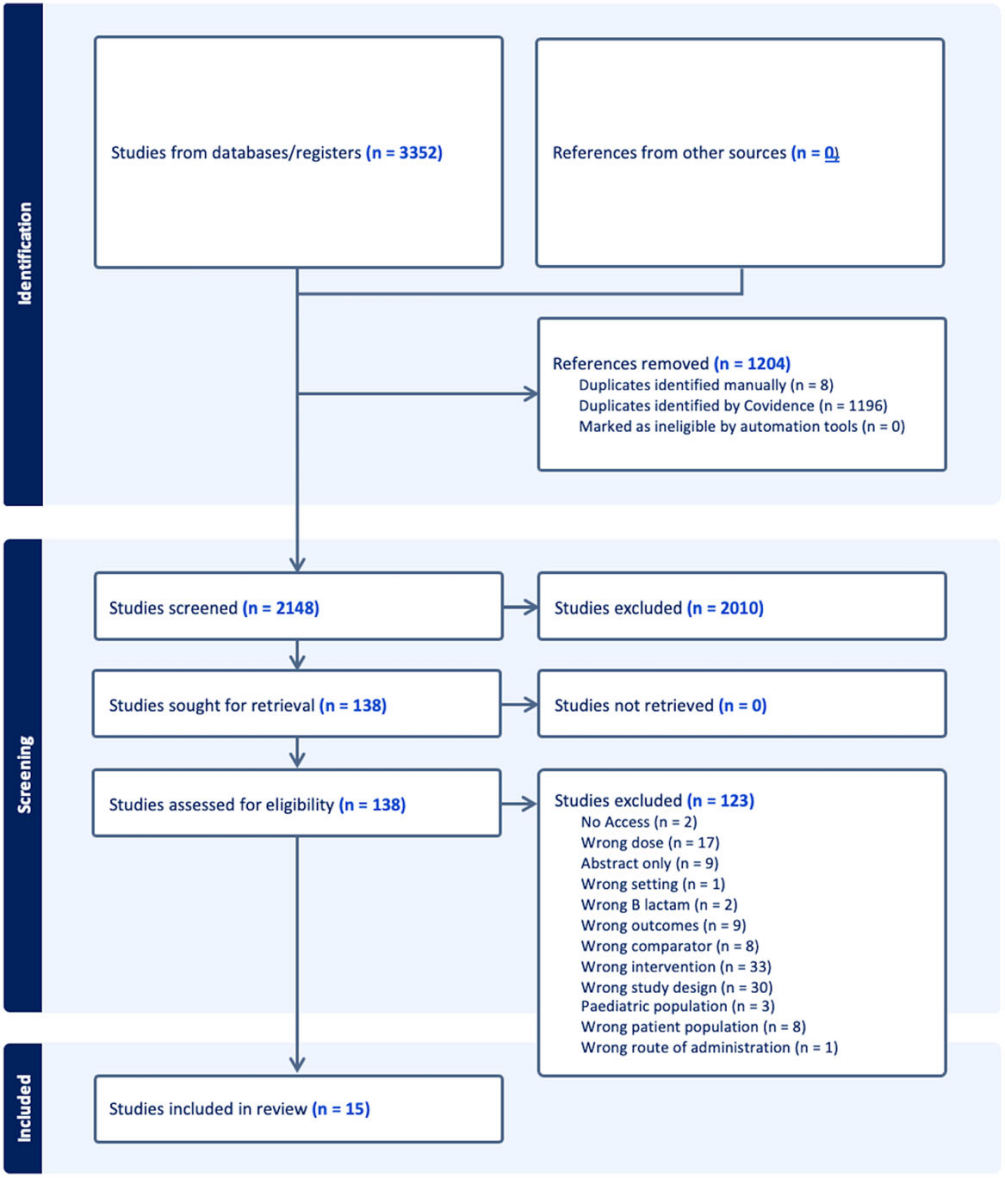
PRISMA flow.

The search yielded 3 352 studies. 1 204 were removed as duplicates. 2 148 were screened at the title and abstract level. 138 were screened at the full-text level. After screening, 15 studies met the inclusion criteria and were included in the analysis (Figure 3). Of these studies, a total of 1786 patients (68%) completed DOPT. There was considerable variation in the number of individual participants in the studies, with a mean of 119 (range 7–675). The 15 studies were conducted in various settings: 5 (33%) in the outpatient setting, 10 (66%) in the inpatient setting, and 1 (7%) in the intensive care unit (ICU), with 2 having multiple settings. Risk stratification scoring systems included the PEN-FAST in 4 (27%), PARSS in 2 (13%), and 9 (60%) used their own definition. DOPT was performed by physicians in 10 (66%), 5 (33%) by pharmacists, 3 (20%) were unknown, and 1 (7%) included advanced practice providers, with 3 studies having multiple types of providers. Overall, 66 (3.7%) patients experienced any reaction following DOPT. Of the 1786 patients who completed DOPT, 66 (3.7%) experienced any reaction: 27 (1.5%) immediate rashes, 24 (1.3%) delayed rashes, and 15 (.8%) other reactions. These reactions included 26 (1.8%) immediate rashes, 13 (.9%) delayed rashes, and 11 (.7%) classified as “other reactions” (Table 1). No cases of anaphylaxis, angioedema, or need for epinephrine were reported. All 15 studies were deemed low risk for bias by JBI criteria (Table 2).

**Table 1.** Summary of included studies

Study	Type of Study	Setting	Screening Tool Used	Provider Performing Testing	Number Tested	Any Reaction (Percent Any Reaction)	Serious Reaction
Stone 2020^14^	Prospective Cohort. Abstract only	ICU	Urticaria only>5 years, GI symptoms only, self limited rash at any point, remote childhood rash with limited details, family history of penicillin allergy, known tolerance of penicillin since reaction	Physician or pharmacist	33	0 (.0%)	0
Pastel 2022^15^	Prospective Cohort. Abstract only	General Internal Medicine Clinic	No history of severe allergy, anaphylaxis, or angioedema	Internist	7	1^#^(14.3%)	0
Lanoue 2022^16^	Prospective Cohort. Abstract only	Inpatient	Reactions greater than 10 years ago that were not severe cutaneous adverse reactions or anaphylaxis	Internist	14	0 (.0%)	0
Rozario 2023^17^	Prospective Cohort	Inpatient	History of cutaneous reactions only more than 1 year ago, or unknown reactions more than 1 year ago	MD, APP, PharmD	81	2^α^ (2.5%)	0
Chua 2023^4^	Prospective Cohort. Abstract only	Inpatient	“standard assessment tool”	Infectious disease	675	27^β^ (4.5%)	0
Denora 2023^18^	Prospective Cohort. Abstract only	Inpatient and outpatient	PARSS	Physician	97	0 (.0%)	0
Trubiano 2022^19^	Prospective Cohort	Inpatient/ICU	Antibiotic Allergy Assessment Tool (AAAT)	Unknown	478	20^π^ (4.2%)	0
Livirya 2022^20^	Prospective cohort	General medicine inpatient ward	Low risk if no: history of a severe cutaneous adverse reaction, interstitial nephritis, vasculitis, hemolytic anemia, anaphylaxis, angioedema, or respiratory symptoms	Unknown	41	0 (.0%)	0
Scanlon 2018^21^	Retrospective cohort. Abstract only	Outpatient	History of benign rash, benign somatic symptoms, or unknown history associated with last penicillin exposure more than 12 months ago	Unknown	25	0 (.0%)	0
Day 2023^22^	Prospective Cohort	Outpatient	PARSS	Physician	12	0 (.0%)	0
Fahmy 2022^23^	Prospective Cohort	Inpatient	PEN-FAST	Pharmacist	11	0 (.0%)	0
Sugrue 2023^24^	Retrospective cohort	Inpatient	PEN-FAST	Infectious disease	46	1^†^ (2.2%)	0
Mitri 2024^25^	Prospective	Inpatient	PEN-FAST	Pharmacist	170	11 (6.5%)*	0
Tsoulis 2024^26^	Prospective	Outpatient	Reaction >10 years ago, onset >1 hour after ingestion, isolated rash without systemic symptoms, no evidence of severe type II-IV reaction, no comorbid cephalosporin allergy.	Internal Medicine	41	4^‡^ (9.8%)	0
Kalra 2025^27^	Prospective	Inpatient	PEN-FAST	Internal Medicine	55	0 (.0%)	0
Total					1786	66 (3.7%)	0

Reported Reactions:

#: mild flushing reaction

α: “delayed urticaria,” maculopapular rash on day 3

β: 14 with an “immunologically mediated reaction;” 1 maculopapular exanthem less than 2 hours after amoxicillin administration; 13 delayed onset maculopapular exanthem.

π: 10 presumed immune-related reactions and 10 non-immune-related reactions (2 itchiness, 4 gastrointestinal, 1 decreased consciousness, 1 self-resolving throat tightness, 1 fever, and 1 unknown)

†: 1 “delayed reaction”

* 7 with delayed onset maculopapular rashes and 4 nonimmune mediated reactions

‡ 2 with flushing sensation and 2 with delayed (days later) benign rash

APP: advanced practice practitioner (NP, PA)

**Table 2.** JBI critical appraisal for cohort

Question	Stone	Pastel	Lanoue	Rozario	Chua	Trubiano	Livirya	Scanlon	Day	Fahmy	Sugrue	Mitri	Tsoulis	Kalra
Is the sample representative of patients in the population as a whole?	Yes	Yes	Yes	Yes	Yes	Yes	Yes	Yes	Yes	Yes	Yes	Yes	Yes	Yes
Are the patients at a similar point in the course of their condition	Yes	Yes	Yes	Yes	Yes	Yes	Yes	Yes	Yes	Yes	Yes	Yes	Yes	Yes
Has bias been minimized in relation to selection of cases?	N/A	N/A	N/A	N/A	N/A	N/A	N/A	N/A	N/A	N/A	N/A	N/A	N/A	N/A
Are founding factors identified and strategies to deal with stated?	N/A	N/A	N/A	N/A	N/A	N/A	N/A	N/A	N/A	N/A	N/A	N/A	N/A	N/A
Are outcomes assessed using objective criteria?	Yes	Yes	Yes	Yes	Yes	Yes	Yes	Yes	Yes	Yes	Yes	Yes	Yes	Yes
Was follow-up carried out within a sufficient period of time?	Yes	Yes	Yes	Yes	Yes	Yes	Yes	Yes	Yes	Yes	Yes	Yes	Yes	Yes
Were the outcomes of people who withdrew described and included in the analysis?	N/A	N/A	N/A	N/A	N/A	N/A	N/A	N/A	N/A	N/A	N/A	N/A	N/A	N/A
Were outcomes measured in a reliable way?	Yes	Yes	Yes	Yes	Yes	Yes	Yes	Yes	Yes	Yes	Yes	Yes	Yes	Yes
Was appropriate statistical analysis used?	Yes	Yes	Yes	Yes	Yes	Yes	Yes	Yes	Yes	Yes	Yes	Yes	Yes	Yes

## Discussion

Penicillin allergy labels (PAL) are ubiquitous and associated with increased morbidity, mortality, and cost. Direct provocation testing is the gold standard for removing or verifying penicillin allergy, as skin testing and in vitro tests alone do not demonstrate 100% sensitivity or specificity.^7^ Challenging low-risk penicillin allergies via single-dose DOPT is safe, as shown by our study of over 1700 patients with zero severe reactions (anaphylaxis, angioedema, need for epinephrine) and a low rate of any reaction (3.7%). This low-risk profile contrasts with the significant risks of maintaining a false penicillin allergy label, such as increased use of broad-spectrum antibiotics, *Clostridium difficile* infections, and increased healthcare costs.^2,3^

Our findings are consistent with a previously published meta-analysis and systematic reviews by Powell, Cooper, and Blumenthal et al, all of which reported that DOPT in low-risk patients was associated with very low rates of severe reactions.^6–8^ Our study adds significant value by focusing solely on a single-dose, nongraded DOPT performed by a nonallergist. Our inclusion and exclusion criteria were intentional. First, we aimed for simplicity. Despite the several well-designed systematic reviews and meta-analyses, the 2022 drug allergy guidelines found insufficient evidence to recommend a particular DPT protocol over another.^10^ Similarly, a 2023 study found ∼ 50% of patients believed their PAL were permanent, while only 4% of primary care providers referred low-risk PAL patients for testing.^11^ Second, we selected the least resource-dependent protocol. In resource-limited areas, a single dose of amoxicillin at 250 mg would enable twice as many patients to undergo DOPT as an amoxicillin 500 mg protocol would. Similarly, the longer the observation period (60 mins vs 120 mins, e.g.,), the fewer patients a single provider can evaluate and the less likely a patient is willing or able to undergo the testing. Thus, at the cost of excluding additional studies and a larger sample size, we gained simplicity, with the hope of improving scalability.

There are several limitations to our study. The primary limitation is the lack of a comparator group. While we demonstrate the safety of single-dose DOPT, we cannot directly compare its effectiveness to other delabeling strategies, such as skin testing followed by oral challenge. When considering this limitation from an effectiveness perspective, it is important to remember that DPT is the gold standard for allergy testing; any patient who undergoes skin testing still requires DPT. Similarly, the recently published PALACE trial mitigates this limitation from a safety perspective.^12^ This randomized controlled trial found no difference in the rate of severe reactions between single-dose DOPT in low-risk patients and those who underwent skin testing + DOPT.^12^ Next, as all of the studies were observational with no comparator, they were at risk for selection bias. The investigators may have consciously or unconsciously selected patients with PALs who were at lower risk than the general population. Lastly, while all the included studies used risk stratification tools with similar criteria, there was no consensus on defining a low-risk patient. Moving forward, we recommend uniformly adopting the PEN-FAST assessment tool, given that it is the only scoring system validated in a randomized controlled trial. Therefore, our findings cannot and should not be extrapolated to high-risk patients for true penicillin allergy.

Despite these limitations, our findings provide compelling evidence for the safety and feasibility of single-dose DOPT by nonallergists in low-risk patients. This approach has the potential to significantly improve penicillin allergy delabeling rates, reduce the burden of false PAL, and improve patient outcomes. Healthcare providers should feel comfortable evaluating and challenging low-risk penicillin allergies using this simplified protocol. Future research should focus on implementing this approach in broader clinical settings, evaluating its cost-effectiveness, and assessing long-term outcomes.
